# Raw starch conversion by *Saccharomyces cerevisiae* expressing *Aspergillus tubingensis* amylases

**DOI:** 10.1186/1754-6834-6-167

**Published:** 2013-11-29

**Authors:** Marko J Viktor, Shaunita H Rose, Willem H van Zyl, Marinda Viljoen-Bloom

**Affiliations:** 1 Department of Microbiology, Stellenbosch University, Private Bag ×1, Stellenbosch, Matieland 7602, South Africa

**Keywords:** Raw starch, Amylolytic yeast, Biofuels, Consolidated bioprocessing, Amylase, *Aspergillus tubingensis*

## Abstract

**Background:**

Starch is one of the most abundant organic polysaccharides available for the production of bio-ethanol as an alternative transport fuel. Cost-effective utilisation of starch requires consolidated bioprocessing (CBP) where a single microorganism can produce the enzymes required for hydrolysis of starch, and also convert the glucose monomers to ethanol.

**Results:**

The *Aspergillus tubingensis* T8.4 α-amylase (*amyA*) and glucoamylase (*glaA*) genes were cloned and expressed in the laboratory strain *Saccharomyces cerevisiae* Y294 and the semi-industrial strain, *S*. *cerevisiae* Mnuα1. The recombinant AmyA and GlaA displayed protein sizes of 110–150 kDa and 90 kDa, respectively, suggesting significant glycosylation in *S*. *cerevisiae*. The Mnuα1[AmyA-GlaA] and Y294[AmyA-GlaA] strains were able to utilise 20 g l^-1^ raw corn starch as sole carbohydrate source, with ethanol titers of 9.03 and 6.67 g l^-1^ (0.038 and 0.028 g l^-1^ h^-1^), respectively, after 10 days. With a substrate load of 200 g l^-1^ raw corn starch, Mnuα1[AmyA-GlaA] yielded 70.07 g l^-1^ ethanol (0.58 g l^-1^ h^-1^) after 120 h of fermentation, whereas Y294[AmyA-GlaA] was less efficient at 43.33 g l^-1^ ethanol (0.36 g l^-1^ h^-1^).

**Conclusions:**

In a semi-industrial amylolytic *S*. *cerevisiae* strain expressing the *A*. *tubingensis* α-amylase and glucoamylase genes, 200 g l^-1^ raw starch was completely hydrolysed (saccharified) in 120 hours with 74% converted to released sugars plus fermentation products and the remainder presumably to biomass. The single-step conversion of raw starch represents significant progress towards the realisation of CBP without the need for any heat pretreatment. Furthermore, the amylases were produced and secreted by the host strain, thus circumventing the need for exogenous amylases.

## Background

Declining oil reserves, political instability, climate change concerns and rising CO_2_ emissions have led to new interest in biofuels to supplement the growing demand for alternative and sustainable sources of transport fuels. Biofuels, which includes bio-ethanol, can be produced from renewable biomass resources that include dedicated crops (e.g. corn), by-products from agricultural processing activities (e.g. sugarcane bagasse) or even organic municipal waste. Although the positive environmental impact and sustainable nature of biofuels render it advantageous over fossil fuels [1], the cost-effective production of biofuels remains a challenge.

Starch, one of the most abundant polysaccharides in nature, has been used for commercial bio-ethanol production for a number of years, with a relatively mature technology developed for corn in the USA [2,3]. The USA produced 52.6 billion litres of ethanol fuel in 2011, an increase from 49.2 billion litres in 2010 [4]. However, the limitations of current starch-to-ethanol processes, in particular the energy-intensive liquefaction and substantial amounts of exogenous amylases required for the subsequent enzymatic hydrolysis to maltose and glucose, significantly impact the economic viability of raw starch as feedstock.

Starch consists of α-1,4 linked glucose units with α-1,6 branching points [5], which require a combination of α-amylases and glucoamylases for complete hydrolysis. The α-amylases (EC 3.2.1.1) hydrolyse the internal α-1,4-bonds of amylose and amylopectin at random, resulting in the production of short polymer chains (dextrins, 10 to 20 glucose units in length) as well as free glucose and maltose units [6]. Glucoamylases (1,4-α-D-glucan glucohydrolase; EC 3.2.1.3) hydrolyse the terminal 1,4-linked α-D-glucopyranosyl units in an exo-fashion successively from the non-reducing end of starch chains to release β-D-glucose [7,8]. When confronted with raw starch, α-amylase will contribute towards the liquefaction of the starch, whilst glucoamylase will predominantly be responsible for the saccharification of the polymers [9,10].

Starch hydrolysing enzymes are abundant in the animal, microbial and plant kingdoms, but only a selected few are able to hydrolyse raw starch [11]. Species of *Aspergillus*, *Fusarium*, *Lipomycetes*, *Mucor*, *Penicillium*, *Rhizopus* and *Rhizomucor* express α- and/or glucoamylases [3,12,13] and some *Aspergillus* and *Rhizopus* spp. have already been exploited for the commercial production of glucoamylases in the food industry [14,15]. Raw starch degrading enzymes (RSDE) that can both liquefy and saccharify raw starch can significantly reduce the energy requirements and simplify the production of starch-based biofuels [16]. However, only a few RSDE have been cloned and characterised, e.g. α-amylases from *Lipomyces kononenkoae *[17], *Streptomyces bovis *[18,19], *Cryptococcus* and *Bacillus *[3], as well as glucoamylases from *Rhizopus oryzae *[18,19] and *Corticium rolfsii *[3].

Cost-effective conversion of raw starch to biofuels requires the expression of starch-hydrolysing enzymes in a fermenting yeast strain to achieve liquefaction, hydrolysis and fermentation (Consolidated Bioprocessing, CBP) by a single organism [11]. The yeast *Saccharomyces cerevisiae* remains the preferred organism for ethanol production due to its high ethanol, osmo- and inhibitor tolerance in industrial processes, but it lacks starch degrading enzymes required for the efficient utilisation of starch [20]. This could potentially be overcome with genetic engineering to allow heterologous expression of the enzymes required for the utilization of starch. Successful expression of recombinant cellulases and hemicellulases in *S*. *cerevisiae* demonstrated the potential of CBP for cellulolytic feedstock [21]. This yeast is therefore the preferred candidate for the construction of an amylolytic yeast strain able to perform CBP of raw starch.

Co-expression of α-amylases and glucoamylases through extracellular secretion or tethering of enzymes on the cell surface of *S*. *cerevisiae* has previously been reported reviewed in [11]. For example, secretion of the *Aspergillus awamori* GA1 and *Debaryomyces occidentalis* AMY in a polyploid *S*. *cerevisiae* was able to convert 98% of 200 g l^-1^ soluble starch to yield 80 g l^-1^ ethanol within 6 days [22]. Although conversion of raw starch by yeast secreting or displaying α-amylases and glucoamylases was previously reported, it involved low substrate loads or conversion rates that will not be economically viable on industrial scale reviewed in [23]. The challenge remains to construct an amylolytic yeast strain that is able to effectively liquefy and saccharify high concentrations of raw starch, as well as ferment the sugars to ethanol within a short time frame.

A screen for potential fungal candidates led to the isolation of an *Aspergillus tubingensis* strain that displayed significant extracellular amylase activity [24]. Although the glucoamylase gene (*glaA*) was previously cloned and sequenced from an *A*. *tubingensis* (*A*. *niger* DSM 823) strain [25], no further characterisation of the protein has been reported. In this study, the α-amylase (*amyA*) and glucoamylase (*glaA*) coding regions were isolated from the *A*. *tubingensis* T8.4 strain and expressed in the laboratory strain *S*. *cerevisiae* Y294 as well as *S*. *cerevisiae* Mnuα1, a semi-industrial strain. The recombinant enzymes were partially characterised and the amylolytic yeast strains evaluated in terms of extracellular amylase activity and conversion of raw starch to ethanol (i.e. consolidated bioprocessing). The co-expressing strains, *S*. *cerevisiae* Y294[AmyA-GlaA] and *S*. *cerevisiae* Mnuα1[AmyA-GlaA], were also evaluated for their ability to hydrolyse and ferment raw starch at a high substrate loading (200 g l^-1^).

## Results and discussion

### Cloning and recombinant expression of *amyA* and *glaA*

Amplification of the *A*. *tubingensis amyA* cDNA yielded a 1 860 base pair (bp) fragment with 99.8% DNA sequence homology to the *A*. *kawachii* α-amylase gene [Genbank: AB008370]. Amplification of the *A*. *tubingensis glaA* cDNA yielded a 1 920 bp fragment with 99.3% DNA homology to the *A*. *shirousami* glucoamylase cDNA [Genbank: D10460 and E02366], as well as 100% homology over 1 917 bp with the *A*. *tubingensis* glucoamylase gene, *glaA* (GenBank: AY528665). The cDNA sequence of the *A*. *tubingensis* α-amylase gene (*amyA*) and predicted amino acid sequence were deposited [GenBank: JF809672].

**Figure 1:**
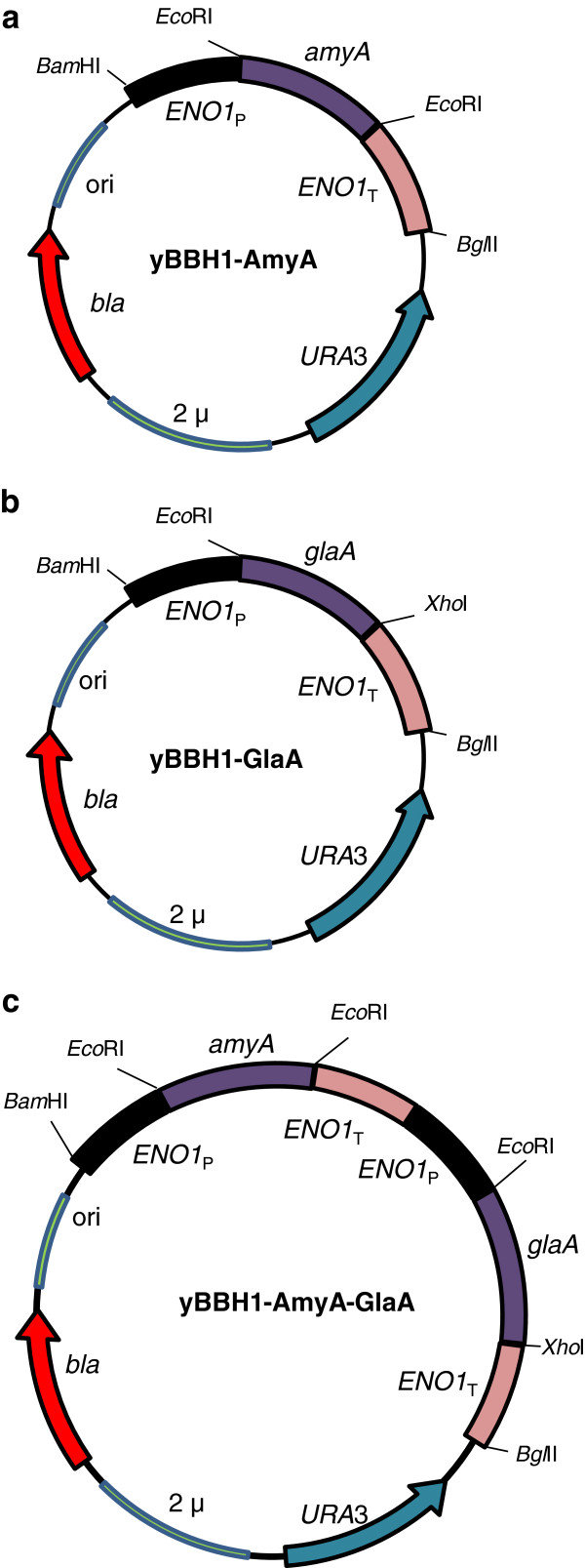
**Schematic representation of the final vector constructs used in this study.** The cDNA of **(a)***amyA* and **(b)***glaA* were expressed in plasmids yBBHI-AmyA and yBBH1-GlaA, respectively, and **(c)** co-expressed in plasmid yBBHI-AmyA-GlaA under regulation of the enolase I (*ENO1*) promoter and terminator sequences. ori, bacterial origin of replication; *bla*, ampicillin-resistance gene; *URA3*, yeast auxotrophic marker; 2 μ, yeast 2-micron origin of replication; *Bam*HI, *Bgl*II, *Eco*RI and *Xho*I, restriction enzyme sites used for cloning.

**Figure 2:**
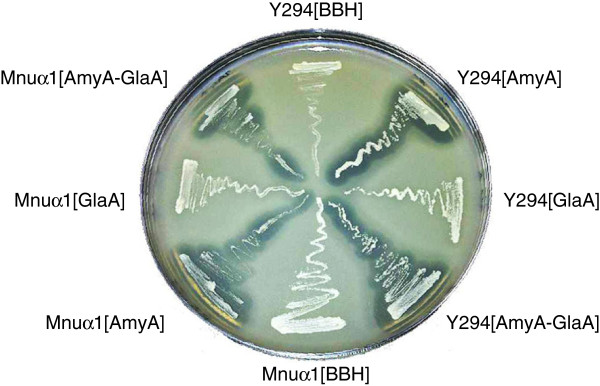
**Plate assays indicate hydrolysis zones surrounding the *S*. *cerevisiae *** **Y294[AmyA], *S*. *cerevisiae *** **Y294[AmyA-GlaA], *S*. *cerevisiae *** **Mnuα1[AmyA] and *S*. *cerevisiae *** **Mnuα1[AmyA-GlaA] strains, whereas the reference strains (*S*. *cerevisiae *** **Y294[BBH1] and *S*. *cerevisiae *** **Mnuα1[BBH1]) and the strains expressing the *glaA *** **(*S*. *cerevisiae *** **Y294[GlaA] and *S*. *cerevisiae *** **Mnuα1[GlaA]) indicated no α-amylase activity.**

The *amyA* and *glaA* genes were subcloned individually and combined in yBBH1-derived plasmids (Figure 1, Table 1) and transformed into the laboratory strain *S*. *cerevisiae* Y294. The *S*. *cerevisiae* strains expressing α-amylase (AmyA) developed clearing zones on solid SC^-URA^ media containing 2% soluble starch (Figure 2), whilst zones were neither expected nor observed for strains expressing the exo-type glucoamylase. Similar results were obtained when the corresponding plasmids were transformed in *S*. *cerevisiae* Mnuα1 strain, a haploid strain derived from the industrial MH1000 strain (Table 1).

**Table 1:** Microbial strains and plasmids used in this study

**Strains or plasmids**	**Genotype**	**Source/reference**
**Strains**		
*E*. *coli* DH5α	*supE44 ΔlacU169* (ϕ*80lacZΔM15*) *hsdR17 recA1 endA1 gyrA96 thi*-*1 relA1*	[26]
*A*. *tubingensis* T8.4	Wild type	[24]
*S*. *cerevisiae* Y294	*α leu2*-*3*,*112 ura3*-*52 his3 trp1*-*289*	ATCC 201160
*S*. *cerevisiae* Y294[BBH1]	*URA3 ENO1*_*P*_-*ENO1*_*T*_	This study
*S*. *cerevisiae* Y294[AmyA]	*URA3 ENO1*_*P*_-*amyA*-*ENO1*_*T*_	This study
*S*. *cerevisiae* Y294[GlaA]	*URA3 ENO1*_*P*_-*glaA*-*ENO1*_*T*_	This study
*S*. *cerevisiae* Y294[AmyA-GlaA]	*URA3 ENO1*_*P*_-*amyA*-*ENO1*_*T*_; *URA3 ENO1*_*P*_-*glaA*-*ENO1*_*T*_	This study
*S*. *cerevisiae* Mnuα1	haploid *ura3, α* derivative of MH1000	[27]
*S*. *cerevisiae* Mnuα1[BBH1]	*URA3 ENO1*_*P*_-*ENO1*_*T*_	This study
*S*. *cerevisiae* Mnuα1[AmyA]	*URA3 ENO1*_*P*_-*amyA*-*ENO1*_*T*_	This study
*S*. *cerevisiae* Mnuα1[GlaA]	*URA3 ENO1*_*P*_-*glaA*-*ENO1*_*T*_	This study
*S*. *cerevisiae* Mnuα1[AmyA-GlaA]	*URA3 ENO1*_*P*_-*amyA*-*ENO1*_*T*_; *URA3 ENO1*_*P*_-*glaA*-*ENO1*_*T*_	This study
**Plasmids**		
pTZ57R/T	*bla*	Fermentas
pTZ-AmyA	*bla amyA*	This study
pTZ-GlaA	*bla glaA*	This study
yBBH1	*bla URA3 ENO1*_*P*_-*ENO1*_*T*_	[28]
yBBH1-AmyA	*bla URA3 ENO1*_*P*_-*amyA*-*ENO1*_*T*_	This study
yBBH1-GlaA	*bla URA3 ENO1*_*P*_-*glaA*-*ENO1*_*T*_	This study
yBBH1-AmyA-GlaA	*bla URA3 ENO1*_*P*_-*amyA*-*ENO1*_*T*_; *ENO1*_*P*_-*glaA*-*ENO1*_*T*_	This study

### Characterisation of recombinant AmyA and GlaA

**Figure 3:**
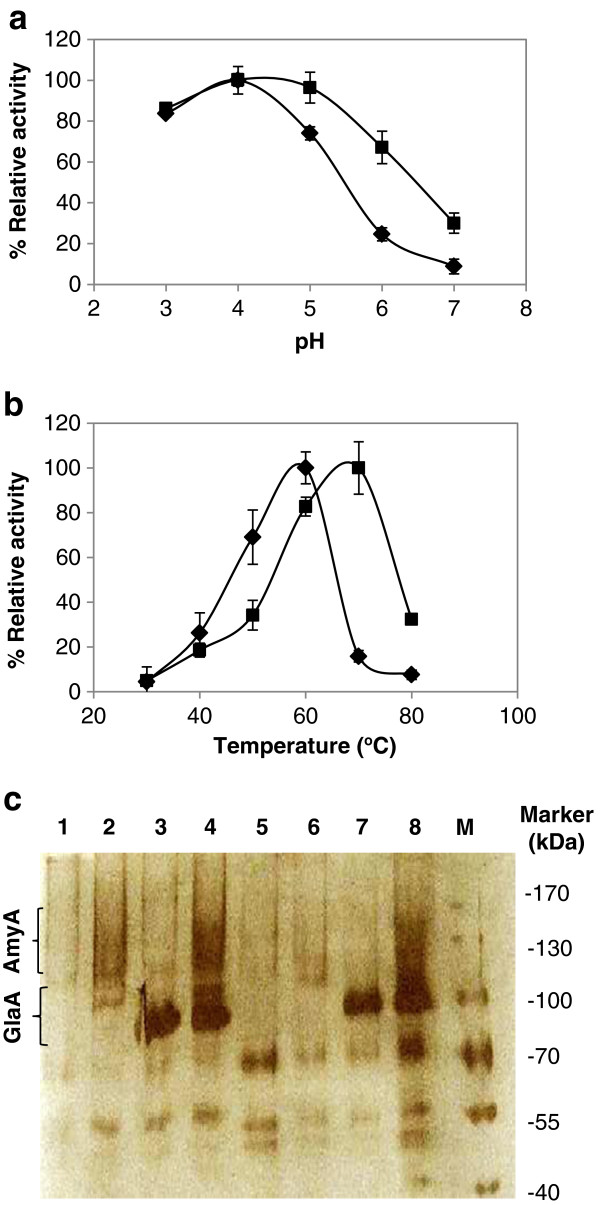
**The relative activity at (a) different pH and (b) temperature levels, and (c) protein size determination of the recombinant enzymes.** (♦) *S*. *cerevisiae* Y294[AmyA] and (■) *S*. *cerevisiae* Y294[GlaA]. Supernatant of *S*. *cerevisiae* Y294[BBH1] (lane 1), *S*. *cerevisiae* Y294[AmyA] (lane 2), *S*. *cerevisiae* Y294[GlaA] (lane 3), *S*. *cerevisiae* Y294[AmyA-GlaA] (lane 4), *S*. *cerevisiae* Mnuα1[BBH1] (lane 5), *S*. *cerevisiae* Mnuα1[AmyA] (lane 6), *S*. *cerevisiae* Mnuα1[GlaA] (lane 7) and *S*. *cerevisiae* Mnuα1[AmyA-GlaA] (lane 8) were subjected to SDS-PAGE followed by silver staining. The protein size marker is depicted on the right hand side.

Maximum activities for the recombinant AmyA and GlaA in *S*. *cerevisiae* Y294 were observed at pH 4.0 and pH 4.5, respectively, with significant activity detected for both enzymes at pH 3 to 5 (Figure 3). These maxima compared well with those reported for other *Aspergillus* α-amylase and glucoamylases [3,16,29] and are aligned with the growth conditions of *S*. *cerevisiae*, which is desirable for a consolidated process [30]. The temperature maxima (Figure 3) for the recombinant AmyA (60°C) and GlaA (70°C) were slightly higher than the 40 to 60°C generally reported for raw starch degrading α-amylases and glucoamylases [3,16,31].

Based on the deduced amino acid sequences, the predicted molecular weights of the unglycosylated AmyA and GlaA were 69.6 kDa and 68 kDa, respectively, which are in agreement with previous reports on similar proteins [25,31,32]. However, SDS-PAGE analysis of the supernatant indicated a large heterogeneous smear between 110 to 150 kDa for all four strains expressing *amyA* (Figure 3c), suggesting differentially glycosylated proteins. The putative recombinant GlaA was observed at approximately 90 kDa, which is within the range reported for fungal glucoamylases [33]. This suggests glycosylation of GlaA, probably at one or more of the eight asparagine-linked glycosylation sites predicted for GlaA [25].

**Figure 4:**
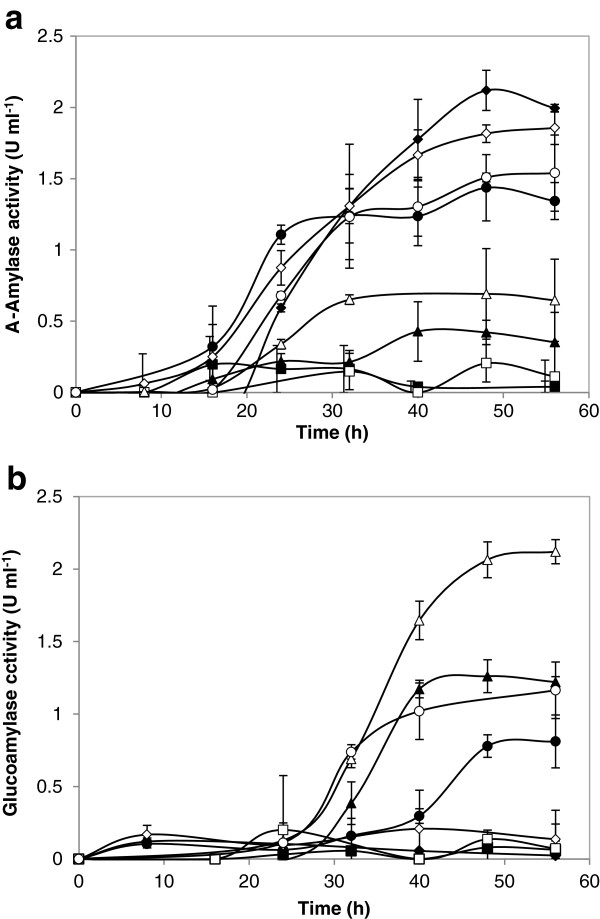
**Extracellular (a) α-amylase and (b) glucoamylase activity determined for (■) *S*. *cerevisiae *Y294[BBH1], (♦) *S*. *cerevisiae *Y294[AmyA], (▲) *S*. *cerevisiae *Y294[GlaA], (●) *S*. *cerevisiae *Y294[Amy-GlaA], (□) *S*. *cerevisiae *Mnuα1[BBH1], (◊) *S*. *cerevisiae *Mnuα1[AmyA], (∆) *S*. *cerevisiae *Mnuα1[GlaA] and (○) *S*. *cerevisiae *Mnuα1[AmyA-GlaA] cultured in double strength SC**^**-URA **^**medium containing 20 g l**^**-1 **^**glucose under aerobic conditions.** Note, (●) and (○) represent the combined α-amylase and glucoamylase activities. Values represent the mean of three repeats and error bars represent the standard deviation.

When cultivated in double strength SC^-URA^ medium with 20 g l^-1^ glucose under aerobic conditions, the extracellular α-amylase activities were similar for *S*. *cerevisiae* Y294[AmyA] and Mnuα1[AmyA] (Figure 4a). However, the glucoamylase activity in the supernatant from *S*. *cerevisiae* Mnuα1[GlaA] was significantly higher than that of *S*. *cerevisiae* Y294[GlaA] (Figure 4b), which could be ascribed to a potentially better secretion ability of *S*. *cerevisiae* Mnuα1. Furthermore, co-production of AmyA and GlaA resulted in lower levels of both activities compared to those observed for the individual enzymes. Similar results were observed for the separate and co-expression of a xylanase and xylosidase in *S*. *cerevisiae* Y294 [34] and could be ascribed to a number of reasons that were not further investigated in this study.

During starch hydrolysis, α-amylases act first to hydrolyse the internal linkages of the starch molecules and thus provide opportunity for saccharification via the glucoamylases, suggesting that production of native α-amylases would precede that of glucoamylases. In the presence study, the activity of the recombinant α-amylase activity increased slightly faster than that of glucoamylase, which is in agreement with the findings of Yamada et al. [18] that the activity of the *S*. *bovis* α-amylase peaked before that of the *R*. *oryzae* glucoamylase when expressed in *S*. *cerevisiae*. The delay may be ascribed to the need for dimerization of the glucoamylase prior to its functioning on insoluble starch, as was described for the *A*. *niger* glucoamylase [35].

### Fermentation of raw starch

**Figure 5:**
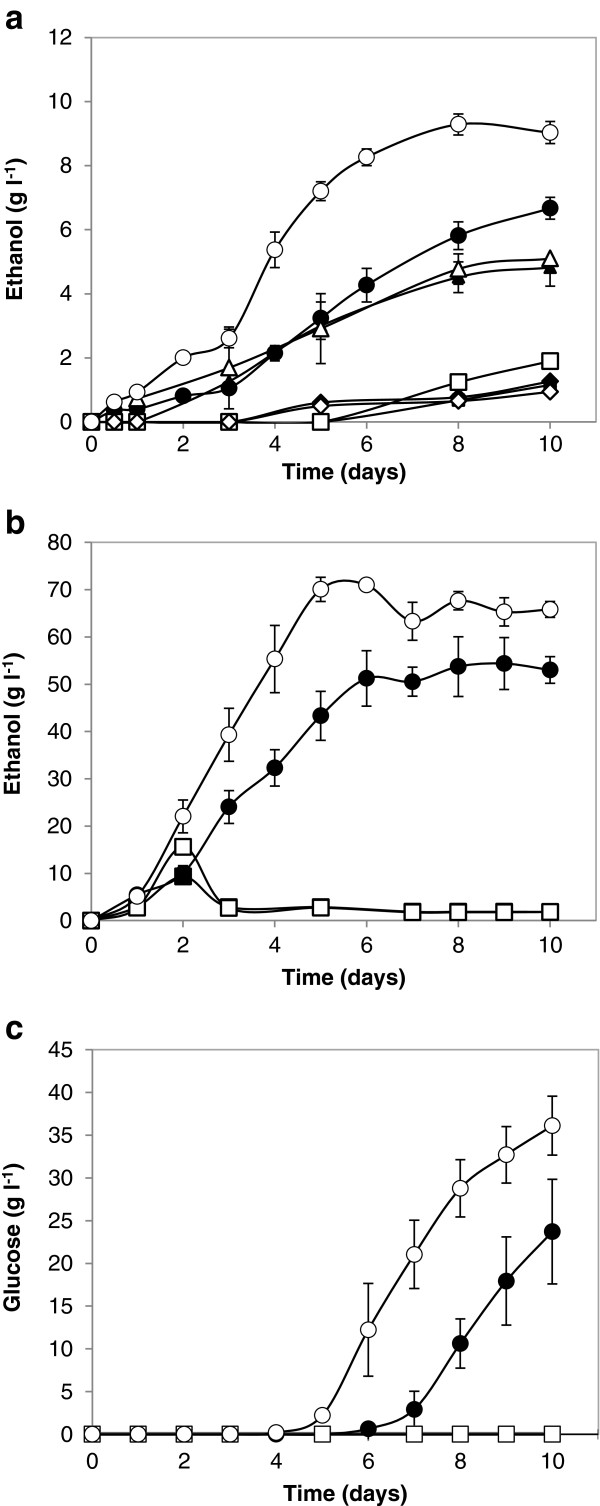
**Ethanol production under oxygen limited conditions in double strength SC**^**-URA **^**media with (a) 20 g l**^**-1 **^**corn starch as sole carbohydrate source, (b) 200 g l**^**-1 **^**corn starch and 5 g l**^**-1 **^**glucose and (c) glucose concentration during growth on 200 g l**^**-1 **^**corn starch and 5 g l**^**-1 **^**glucose.** (■) *S*. *cerevisiae* Y294[BBH1], (♦) *S*. *cerevisiae* Y294[AmyA], (▲) *S*. *cerevisiae* Y294[GlaA], (●) *S*. *cerevisiae* Y294[Amy-GlaA], (□) *S*. *cerevisiae* Mnuα1[BBH1], (◊) *S*. *cerevisiae* Mnuα1[AmyA], (∆) *S*. *cerevisiae* Mnuα1[GlaA] and (○) *S*. *cerevisiae* Mnuα1[AmyA-GlaA]. Values represent the mean of three repeats and error bars represent the standard deviation. Note, some data points may overlap, in particular for the control strains.

After 10 days of cultivation on 20 g l^-1^ raw corn starch as sole carbohydrate source under fermentative conditions, simultaneous expression of the *A*. *tubingensis* α-amylase and glucoamylase in *S*. *cerevisiae* resulted in ethanol yields of 6.67 g l^-1^ and 9.03 g l^-1^ by *S*. *cerevisiae* Y294[AmyA-GlaA] and *S*. *cerevisiae* Mnuα1[AmyA-GlaA], respectively (Figure 5). The [AmyA] and [GlaA] strains converted less than 15% and 50% of the available carbon, respectively, whereas the [AmyA-GlaA] strains exceeded a 70% carbon conversion rate (Table 2). This confirmed that both the α-amylase and glucoamylase are required for efficient ethanol production from raw corn starch. Given the substrate loading of 20 g l^-1^ raw starch, a yield of 9.03 g l^-1^ ethanol for *S*. *cerevisiae* Mnuα1[AmyA-GlaA] translated into 83% carbon conversion and 80% of the theoretical ethanol yield (Table 2). This was statistically significantly higher than the ethanol yield from *S*. *cerevisiae* Y294[AmyA-GlaA] and represented a 4.76-fold improvement relative to the parental *S*. *cerevisiae* Mnuα1[BBH1] strain.

**Table 2:** **Conversion of raw starch to ethanol and byproducts by recombinant *S*. *cerevisiae *** **strains**

**Substrate/product**	***S*. *cerevisiae *** **Y294**					***S*. *cerevisiae *** **Mnuα1**				
**(g l**^**-1**^**)**	**[AmyA]**	**[GlaA]**	**[AmyA-GlaA]**			**[AmyA]**	**[GlaA]**	**[AmyA-GlaA]**		
	***Day 10***	***Day 10***	***Day 10***	***Day 5***	***Day 10***	***Day 10***	***Day 10***	***Day 10***	***Day 5***	***Day 10***
***Substrate***										
Raw starch	20.0	20.0	20.0	200.0	200.0	20.0	20.0	20.0	200.0	200.0
Glucose				5.0	5.0				5.0	5.0
*Glucose equivalent*	*22*.*2*	*22*.*2*	*22*.*2*	*227*.*0*	*227*.*0*	*22*.*2*	*22*.*2*	*22*.*2*	*227*.*0*	*227*.*0*
***Products***										
Glucose	ND	ND	0.31 ± 0.06	ND	23.71 ± 6.13	ND	ND	ND	2.21 ± 0.40	36.11 ± 3.44
Maltose	ND	ND	ND	0.39 ± 0.04	ND	ND	ND	ND	0.37 ± 0.04	ND
Glycerol	0.12 ± 0.01	0.89 ± 0.18	1.50 ± 0.17	3.17 ± 0.32	3.1 ± 0.13	ND	0.19 ± 0.03	0.42 ± 0.01	3.93 ± 0.09	2.63 ± 0.09
Acetic acid	0.32 ± 0.01	0.59 ± 0.07	0.69 ± 0.07	0.65 ± 0.04	0.14 ± 0.04	0.32 ± 0.01	0.36 ± 0.02	0.3 ± 0.01	0.40 ± 0.03	ND
Ethanol	1.27 ± 0.03	4.82 ± 0.58	6.67 ± 0.65	43.33 ± 5.20	53.02 ± 2.80	0.94 ± 0.15	5.10 ± 1.03	9.03 ± 0.34	70.07 ± 2.58	65.83 ± 1.67
CO_2_	1.22	4.61	6.38	41.45	50.71	0.90	4.88	8.64	67.02	62.97
**Total carbon**	**2.94**	**10.92**	**15.55**	**88.98**	**130.68**	**2.16**	**10.52**	**18.45**	**144.00**	**167.54**
Carbon conversion rate	13%	49%	70%	39%	58%	10%	47%	83%	63%	74%
Ethanol (% theoretical)	11%	43%	59%	37%	46%	8%	45%	80%	61%	57%
Ethanol productivity	0.01	0.02	0.03	0.36	0.22	0.00	0.02	0.04	0.58	0.27

ND, not detected.

Towards the end of the fermentation, 0.31 g l^-1^ residual glucose was present in the Y294[AmyA-GlaA] culture, suggesting that fermentation could be rate-limiting for the Y294 strain. The *S*. *cerevisiae* Y294[AmyA] strain displayed some accumulation of maltose (0.42 and 0.69 g l^-1^ on day 5 and 8, respectively), whereas the Y294[AmyA-GlaA] metabolised the maltose much quicker (decreasing from 0.33 g l^-1^ on day 5 to 0.06 g l^-1^ on day 8) due to the presence of the glucoamylase. In contrast, the Mnuα1 strain has a native maltase, with no interim maltose accumulation observed for the any of the respective strains (data not shown). The recombinant *S*. *cerevisiae* Y294 strains produced more acetic acid and glycerol than the Mnuα1 strains, suggesting that the Y294 strains were coping less effectively with the starch fermentation conditions.

The CBP simulation was performed under fermentative conditions with *S*. *cerevisiae* Y294[AmyA-GlaA], *S*. *cerevisiae* Mnuα1[AmyA-GlaA] and the corresponding control strains using 200 g l^-1^ raw starch as well as 5 g l^-1^ glucose to provide an initial carbon source to the cells. The *S*. *cerevisiae* Y294[AmyA-GlaA] and Mnuα1[AmyA-GlaA] strains produced 43.33 and 70.07 g l^-1^ ethanol, respectively (corresponding to 37% and 61% of the theoretical yield) after 5 days of fermentation (Table 2). Although the ethanol concentration did not increase significantly after day 5, glucose accumulation in both strains indicated continued saccharification of the remaining starch (Figure 5). Glucose accumulation in the *S*. *cerevisiae* Mnuα1[AmyA-GlaA] fermentation (2.21 and 36.11 g l^-1^ after 5 and 10 days, respectively) suggested that the fermentation capability of the strain became the limiting factor. The glucose accumulation was less significant for *S*. *cerevisiae* Y294[AmyA-GlaA], with only 23.71 g l^-1^ glucose detected after 10 days. However, the activity of the recombinant GlaA in particular was significantly lower in the Y294 strain (Figure 4), which will reduce its saccharification ability relative to that of the *S*. *cerevisiae* Mnuα1[AmyA-GlaA] strain.

## Conclusions

Co-expression of the *A*. *tubingensis amyA* and *glaA* genes on episomal plasmids conveyed amylolytic activity to both a laboratory (Y294) and a semi-industrial strain (Mnuα1) of *S*. *cerevisiae*. The α-amylase and glucoamylase activities reached 1.51 and 1.16 U ml^-1^, respectively, in the Mnuα1[AmyA-GlaA] strain, which compare favourably with the 96–190 U ml^-1^ and 140–340 U ml^-1^ reported previously for α-amylase and glucoamylase expression in other haploid strains [18].

The recombinant *S*. *cerevisiae* Mnuα1[AmyA-GlaA] strain was superior in its ability to convert 83% of the available carbon in 20 g l^-1^ raw corn starch and produced 80% of the theoretical ethanol yield after 10 days. At a higher substrate loading of 200 g l^-1^ raw corn starch, 61% and 57% of the theoretical ethanol yield was achieved within 5 and 10 days, respectively. The starch was completely hydrolysed (saccharified) with 74% converted to released sugars plus fermentation products (mainly ethanol, glycerol and CO_2_) and the remainder presumably to yeast biomass. The lower ethanol and residual glucose levels for the *S*. *cerevisiae* Y294[AmyA-GlaA] fermentation suggested weaker saccharification by the recombinant *S*. *cerevisiae* Y294 strain, whereas fermentation capacity is the limiting factor for the *S*. *cerevisiae* Mnuα1[AmyA-GlaA] strain.

As different experimental procedures were used in other reports on raw starch-degrading yeasts, it is difficult to compare the results from the present study with those previously reported. The *S*. *cerevisiae* YF237 strain, displaying the *Rhizopus oryzae* glucoamylase and secreting the *Streptococcus bovis* α-amylase, was reported to produce 51 g l^-1^ of ethanol from 100 g l^-1^ of raw corn starch after 60 h of fermentation [36]. A diploid strain displaying both these proteins on the cell surface, produced 46.5 g l^-1^ of ethanol from 200 g l^-1^ raw corn starch after 120 h of fermentation [37], i.e. an ethanol productivity of 0.43 g l^-1^ h^-1^. The *S*. *cerevisiae* Mnuα1[AmyA-GlaA] strain produced 70.07 g l^-1^ of ethanol from 200 g l^-1^ of raw corn starch after 120 h of fermentation (i.e. an ethanol productivity of 0.58 g l^-1^ h^-1^), which is significantly higher than that reported for the diploid strain mentioned above. Also, in contrast to the previously mentioned studies, the enzymes in this study were not tethered to the cell wall of precultured cells, but were both produced and secreted during cultivation on raw corn starch.

Bio-ethanol production from starch substrates has surpassed that of sugarcane in recent years and will still play a major role in years to come. Starch is much more readily degradable relative to cellulosic material, which is much more recalcitrant by nature. However, the production of ethanol from starch should not be seen as a “stand alone” option that could potentially threaten food security [38], but rather as part of an integrated bio-ethanol industry that utilise both starchy and cellulosic feedstocks. More cost-effective starch utilization processes could be implemented when it forms part of a biorefinery concept for whole plant utilisation, which will ultimately contribute to optimum biomass conversion and increased energy efficiency [39,40]. The single-step conversion of raw starch to ethanol represents significant progress towards the realisation of consolidated bioprocessing without the need for heat pretreatment or exogenous enzymes. Taking into consideration that these were small-scale fermentation studies with no process optimisation, the current performance of the Mnuα1[AmyA-GlaA] strain warrants further development, including chromosomal integration of *amyA* and *glaA* in a yeast strain with a stronger fermentation capacity. Furthermore, repeated fermentations will most likely further improve the efficiency of the Mnuα1[AmyA-GlaA] strain, as was previously reported for other amylolytic strains that reached ethanol productivity and yield of 1.61 g l^-1^ h^-1^ and 76.6% after 23 cycles [37].

## Methods

### Strains and media

All strains and plasmids used in the study are listed in Table 1. The *A*. *tubingensis* T8.4 strain is protected under patent no. WO/2011/128712 and was deposited in the South African Plant Protection Research Institute’s culture collection [PPRI 13401].

All chemicals, media components and supplements were of analytical grade. Recombinant plasmids were constructed and amplified in *Escherichia coli* DH5α, cultivated aerobically at 37°C in Terrific Broth or on Luria Bertani agar [26], containing 100 μg/ml ampicillin when required.

The *A*. *tubingensis* T8.4 strain was maintained on MEA plates (50 g l^-1^ malt extract agar, Sigma-Aldrich) at 30°C. For cDNA preparation, *A*. *tubingensis* was cultivated in liquid synthetic complete (SC) medium (1.7 g l^-1^ yeast nitrogen base without amino acids, Difco Laboratories) with 2% raw corn starch (Sigma-Aldrich) in 125 ml Erlenmeyer flasks for 3 days at 100 rpm.

The *S*. *cerevisiae* Y294 and Mnuα1 host strains were cultivated in YPD medium (10 g l^-1^ yeast extract, 20 g l^-1^ peptone and 20 g l^-1^ glucose). Yeast transformants were selected and maintained on SC medium supplemented with amino acids excluding uracil (SC^-URA^). Aerobic cultivation was performed in 125 ml Erlenmeyer flasks containing 20 ml SC^-URA^ medium on a rotary shaker at 200 rpm at 30°C, unless stated otherwise.

For fermentation studies, pre-cultures were prepared in double strength SC^-URA^ media and transferred to 120 ml glass serum bottles (in triplicate) containing double strength SC^-URA^ media with 20 g l^-1^ raw corn starch as sole carbohydrate source. For the higher substrate loading, pre-cultures were transferred to 120 ml glass serum bottles (in triplicate) containing double strength SC^-URA^ media with 200 g l^-1^ raw corn starch and 5 g l^-1^ glucose.

### DNA amplification and sequence analyses

Table 3 lists the primers (Integrated DNA Technologies) used for PCR amplification of the respective genes. Primers AmyA-L + AmyA-R were based on the cDNA sequence of the *Aspergillus kawachii* amylase gene [GenBank: AB008370], while the GlaA-L + GlaA-R primers were based on the cDNA sequences of the *Aspergillus shirousami* glucoamylase gene [GenBank: D10460 and E02366]. The *A*. *tubingensis* species was verified with PCR amplification of the internal transcribed spacer (ITS) region of its genomic DNA using the universal ITS1 and ITS4 primers [27].

**Table 3:** **PCR primers used in the study with the relevant restriction sites in italics *(Eco*** **RI *= GAATTC; Xho*** **I *= CTCGAG)***

**Primer name**	**Sequence (5’-3’)**	**Reference**
cDNA *amyA*		[GenBank: AB008370]
AmyA-L	AA*GAATTC*CGCTTCGCCAAG	
AmyA-R	CT *GAATTCCTCGAG*ATCAACCACCGTC	
cDNA *glaA*		[GenBank: D10460]
GlaA-L	CA*GAATTC*CACCGCAATGTCGTTC	
GlaA-R	AG*CTCGAG*AATAGTCTACCGCCAGGT	
Identification		[41]
ITS1	TCCGTAGGTGAACCTTGCGG	
ITS4	TCCTCCGCTTATTGATATGC	

Total nucleic acid was isolated from *A*. *tubingensis* (grown in SC with 2% raw corn starch) using liquid nitrogen [42] and mRNA was retrieved with the FastTrack 2.0 mRNA Isolation Kit (Invitrogen Corporation, Carlsbad, CA, USA). First strand cDNA was obtained with the RevertAid™ H Minus First Strand cDNA Synthesis Kit (ThermoScientific, South Africa) and used for amplification of the complete cDNA copies of *amyA* and *glaA* using a Perkin Elmer Gene Amp® PCR System 2400 and TaKaRa Ex Taq™ (Takara Bio Inc, Japan) as per the manufacturer’s recommendations. The *amyA* and *glaA* cDNA fragments were blunt-end ligated into the pTZ57R/T vector (InsTAclone™ PCR Cloning Kit, ThermoScientific), thereafter designated pTZ-AmyA and pTZ-GlaA, respectively. Sequence analysis was done with the ABI PRISM™ 3100 Genetic Analyser, BLAST program (http://blast.ncbi.nlm.nih.gov/Blast.cgi) and DNAMAN (version 4.1) (Lynnon Biosoft).

### DNA manipulation

Standard protocols were followed for DNA manipulation [26] with enzymes for restriction digests and ligations sourced from Roche Applied Science (Germany). Where applicable, DNA was eluted from agarose gels with the Zymoclean™ Gel Recovery Kit (Zymo Research). The *glaA* gene was subcloned as an *Eco*RI-*Xho*I fragment and the *amyA* gene as an *Eco*RI fragment into the corresponding sites of plasmid yBBH1, yielding plasmids yBBH1-AmyA and yBBH1-GlaA, respectively (Figure 1). The *ENO1*_*P*_-*GlaA*-*ENO1*_*T*_ cassette was excised from yBBH1-GlaA as a *Bam*HI-*Bgl*II fragment and cloned into the *Bgl*II site on pBBH1-AmyA, generating pBBH1-AmyA-GlaA (Figure 1).

The host strains, *S*. *cerevisiae* Y294 and *S*. *cerevisiae* Mnuα1, were transformed with the recombinant plasmids using electroporation [43] with subsequent selection on SC^-URA^ plates. The presence of the respective amylase genes was verified by PCR amplification with gene-specific primers (Table 3).

### Amylase assays

For qualitative assays, recombinant *S*. *cerevisiae* strains were cultured on SC^-URA^ plates containing 2% soluble corn starch for 48 hours at 30°C. The plates were transferred to 4°C to allow precipitation of the residual starch, resulting in clear zones around colonies secreting α-amylases.

For quantitative assays, yeast transformants were cultured in 20 ml double-strength SC^-URA^ medium in 125 ml Erlenmeyer flasks for 3 days with agitation at 200 rpm. The supernatant was harvested and enzyme activity levels were determined after 5 minutes with the reducing sugar assay [44] in citrate-phosphate buffer containing 0.2% soluble corn starch at 30°C and pH 5. For glucoamylase activity, 50 μl supernatant was incubated for 5 minutes with 450 μl of a 0.2% soluble corn starch solution (30°C, pH 5). The resulting glucose concentration was determined with the D-Glucose Assay Kit (Megazyme, Ireland). Enzyme activity was expressed as U ml^-1^ supernatant, with one unit defined as the amount of enzyme required to release 1 μmole of glucose per minute. The assays were repeated for *S*. *cerevisiae* Y294[AmyA] and Y294[GlaA] at different pH (pH 3, 4, 5, 6 and 7) and temperature values (30°C, 40°C, 50°C, 60°C, 70°C and 80°C).

### Protein analyses

Recombinant *S*. *cerevisiae* strains were cultivated in 20 ml SC^-URA^ medium for 3 days. Two micrograms of lyophilised supernatant were resuspended in 20 μl distilled water, loading buffer was added and the samples denatured by boiling for 3 minutes. The recombinant enzymes were separated on an 8% SDS-polyacrylamide gel using a 5% stacking gel and Tris-glycine buffer [26]. Electrophoresis was carried out at 100 V for ± 90 minutes at ambient temperature and protein species were visualised with the silver staining method [45].

### Fermentation studies

The precultures were inoculated into double strength SC^-URA^ media with the appropriate carbon sources to a final concentration of 1×10^6^ cells ml^-1^. Ampicillin (100 μg ml^-1^) and streptomycin (15 μg ml^-1^) were added to inhibit bacterial contamination. Agitation and incubation were performed on a magnetic multi-stirrer at 30°C, with regular sampling through a syringe needle pierced through the rubber stopper. Fermentation with high substrate loading was performed similarly, but the double strength SC^-URA^ media containing 200 g l^-1^ raw corn starch and 5 g l^-1^ glucose, was inoculated with a 50 g l^-1^ inoculum (wet weight). The wet cell weight was determined by weighing a cell pellet obtained from centrifugation of the pre-culture at 3 000 × g for 5 minutes.

Ethanol, glycerol, acetic acid, maltose and glucose concentrations were quantified with HPLC, using a Surveyor Plus liquid chromatograph (Thermo Scientific) consisting of a liquid chromatography pump, autosampler and Refractive Index Detector. The compounds were separated on a Rezex RHM Monosaccharide 7.8 × 300 mm column (00H0132-K0, Phenomenex) at 60°C with 5 mM H_2_SO_4_ as mobile phase at a flow rate of 0.6 ml min^-1^. The theoretical CO_2_ yields were calculated based on the ethanol concentrations.

## Abbreviations

CBP: Consolidated bioprocessing; RSDE: Raw starch degrading enzymes; bp: Base pair; MEA: Malt extract agar; SC: Synthetic complete; SC-URA: Synthetic complete lacking uracil; YPD: Yeast extract, peptone, dextrose; DNA: Deoxyribonucleic acid; cDNA: Copy-deoxyribonucleic acid; mRNA: Messenger-ribonucleic acid; PCR: Polymerase chain reaction; ITS: Internal transcribed spacer; SDS-PAGE: Sodium dodecyl sulphate polyacrylamide gel electrophoresis; BLAST: Basic local alignment search tool; HPLC: High performance liquid chromatography; ND: Not detected.

## Competing interests

The authors declare that they have no competing interests.

## Authors’ contributions

MJV participated in the design of the study, performed the experimental work and analyses related to the cloning and characterisation in Y294, and drafted the manuscript. SHR performed the experimental work and analyses related to subcloning and expression in Mnuα1, participated in the design of the study and revised the manuscript. WHVZ and MVB participated in the design of the study and revised the manuscript. All authors read and approved the final manuscript.
